# Designing novel construction for cell surface display of protein E on *Escherichia coli* using non-classical pathway based on Lpp-OmpA

**DOI:** 10.1186/s13568-017-0350-0

**Published:** 2017-02-28

**Authors:** Meisam Jeiranikhameneh, Mohamad Reza Razavi, Shiva Irani, Seyed Davar Siadat, Mana Oloomi

**Affiliations:** 1 Department of Biology, School of Basic Science, Science and Research Branch, Islamic Azad University, Tehran, Iran; 2 Molecular Parasitology Laboratory, Department of Parasitology, Pasteur Institute of Iran, 69 Pasteur Avenue, Tehran, 1316943551 Iran; 3 Department of Tuberculosis and Pulmonary Research, Pasteur Institute of Iran, Tehran, Iran; 4 Department of Molecular Biology, Pasteur Institute of Iran, Tehran, Iran

**Keywords:** Surface display, Non-classical secretion pathway, Lpp-OmpA, *Haemophilus influenza*, Protein E

## Abstract

Today, transference of recombinant protein on the outer surface of bacteria is deemed as a valuable process for various applications in biotechnology including preparation of vaccines. In this study, Lpp-OmpA structure was used to present outer membrane protein E of *Haemophilus influenzae* on *E. coli* outer membrane. Also, a structure was designed according to Lpp-OmpA based on non-classical secretion pathway using bioinformatics software such as MEMSAT-SVM, ScrotumP and SignalP where it lacked any signal peptide at its N-terminal. Potential of this structure in the presentation of protein E on the surface of *E. coli* through non-classical pathway was indicated by western blotting, SDS page and fluorescent microscopy techniques, similarly its effectiveness was compared with Lpp-OmpA system. The results of the current study showed that the new structure had higher efficiency than Lpp-OmpA, and it could transport protein E on outer membrane well. This study is the first report in the presentation of *H. influenzae* PE onto the surface of *E. coli* by Lpp-OmpA, and the structure originated from Lpp-OmpA, according to the non-classical secretion pathway. Our results suggest that non-classical secretion pathway may be exploited as a new secretory pathway on the outer surface of the cell for recombinant proteins.

**Electronic supplementary material:**

The online version of this article (doi:10.1186/s13568-017-0350-0) contains supplementary material, which is available to authorized users.

## Introduction

*Haemophilus influenzae* is a significant pathogen that is categorized into two different main groups predicated on the presentation of a polysaccharide capsule. Encapsulated *H. influenzae* has been classified to six various serotypes (a–f), the large number of them are the reason to invasive sickness, and the nonencapsulated *H. influenzaee* has been determined nontypeable *H. influenzaee* (NTHi), are considered as accountable for the preponderance of mucosal Haemophilus putridity and infections in the respiratory tract (Turk 1984; Meats et al. 2003; Pittman 1931).

Protein E is a 16 kDa outer membrane lipoprotein observed in both enclosed NTHi and *H. influenzae* with sticky attributes (Ronander et al. 2008). The *pe* gene is necessarily expressed in NTHi and is extremely protected between clinical NTHi isolates. The central sector of the molecule (PE84-108) has been specified to act as an active place for the interplay with epithelial cells (Hallstrom et al. 2009; Singh et al. 2010). Besides, PE interlocks human vitronectin and laminin, which indicates that it is multipurpose. Protein E has the capability to be appropriate as a vaccine candidate, because it is available everywhere, exceedingly conserved nature, preferred immunogenicity, and played a preservative role in model animals (Ronander et al. 2009; Singh et al. 2013).

Heterologous proteins on the external surface of microorganisms have been enabled with the aid of recombinant DNA technology and employed strategy in different applications in biotechnology, microbiology, and vaccinology more and more (Chen and Georgiou 2002; Choi and Lee 2004). Several surface-anchoring patterns that have potential to transport to the outer membrane that engaged to the aim of chimeric proteins onto the cell surface, like the lipoprotein outer membrane protein A (Lpp-OmpA) and ice nucleation protein (INP) (Shimazu et al. 2001; Lee et al. 2003).

The output and performance of surface display systems are extremely relevant to the carrier and passenger protein characteristics, host cell, and fusion manner. Lpp-OmpA system that incorporate the interests of proper surface display of outer membrane proteins and provides C-terminal fusions to be created by Georgiou and coworkers (Georgiou et al. 1996). The Lpp-OmpA includes a lipoprotein signal peptide and the first nine N-terminal amino acids of the *E. coli* lipoprotein (Lpp) attached to a transmembrane domain (amino acids 46–159) from outer membrane protein A (OmpA) (Francisco et al. 1993). The Lpp-OmpA-based cell display system was the first prosperous method for displaying full-length heterologous proteins on the *E. coli* surface and was widely applied for heterologous proteins display, like β -lactamase, cellulases, the scFv antibody, cyclodextrin glucanotransferase, the cellulose-binding domain and the chitin-binding domain, on the *E. coli* surface (Georgiou et al. 1997; Francisco et al. 1992, 1993). Nevertheless, it seems to be susceptible to passenger’s secondary and tertiary construction are the chief drawback of this system (Stathopoulos et al. 1996).

Some bacterial proteins have been observed to be secreted without any apparent signal peptide. In this system, known as non-classical secretion, protein secretion does not depend on any of the five recognized secretion pathways (type I–V) in Gram-negative bacteria (Bendtsen et al. 2005; Choi and Lee 2004). The non-classical protein secretion pathway may be exploited as a new secretion pathway for recombinant protein, and is a perfect supplement to the classical secretion pathway (Wang et al. 2015). In the present study, the constructed chimeric protein applied this system to transfer heterologous protein to *E. coli* outer cell surface and its efficiency was forecasted by some prediction software tools as SignalP and SecretomeP. Here, a new anchor system is developed to display by fused OmpA (amino acids 46–159) devoid of Lpp and any signal peptide with protein E termed Non-OmpA-PE. Also, this chimeric protein is aimed and joined to *E. coli* outer cell surface and contrasted its surface targeting efficiency with the Lpp-OmpA system.

## Materials and methods

### Strains, plasmids and culture conditions

All strains, plasmids and primers used in this study are listed in Table 1. Nontypeable *H. influenzae* ATCC 49766 was grown on chocolate agar plates at 37 °C and then in Luria–Bertani broth medium, supplemented with V factor (NAD) and X-factor (hemin-l-histidine) in a humid atmosphere containing 5% CO_2_. *E. coli* Dh5α was employed as a recombinant host for sub cloning and plasmid manipulation, while *E. coli* strain BL21 (DE3) was served as a host for gene expression and surface display of Lpp-OmpA-PE and Non-OmpA-PE. Recombinant cells were grown in Luria–Bertani media at 37 °C supplemented with 50 µg/ml kanamycin.

**Table 1:** Strains, plasmids and primers used in this study

**Strains, plasmids** and primers	Description	Source
Strains		
*E. coli* DH5α	F^−^ Φ80*lac*ZΔM15 Δ(*lac*ZYA-*arg*F) U169 *rec*A1 *end*A1 *hsd*R17 (rk^−^, mk^+^) *pho*A *sup*E44 λ^−^ *thi* ^−^1 *gyr*A96 *rel*A1	Invitrogen
*E. coli* BL21 DE3	F^−^ompTgaldcmlonhsdSB(rB-mB-)λ(DE3[lacIlacUV5-T7gene1ind1sam7nin5], expression host	Stratagene
Nontypeable *H. influenza* ATCC 49766	The source of gene for protein E	Pasteur Institute of Iran
Plasmids		
pET-26b	T7promoter, an N-terminal pelB signal sequence for potential periplasmic localization, plus optional C-terminal His Tag sequence, kanamycin resistance	Novagen
pET-21b	T7promoter, an N-terminal T7 Tag sequence plus an optional C-terminal His Tag sequence, ampicillin resistance	Novagen
pGEM-JR	The source of gene for Lpp-OmpA	Bioneer
p21PE	Expression vector; Amp^r^	This study
pPE22-160	Expression vector; Kan^r^	This study
pLpp-OmpA	Expression vector; Kan^r^	This study
pNon-OmpA	Expression vector; Kan^r^	This study
pLpp-OmpA-PE	Expression vector; Kan^r^	This study
pNon-OmpA-PE	Expression vector; Kan^r^	This study

Primers	Sequence (5′–3′)	Restriction enzymes
*Eco*RIpE22	GTCAGAATTCAAAGGCTAAACAAAATGATGT	*Eco*RI
*Hin*dIIIpE22	GATCAAGCTTTTTTTTATCAACTGAAAATGC	*Hin*dIII
*Nco*IpE22	CATGCCATGGATAAGGCTAAACAAAATGATGTG	*Nco*I
PENdeI	GGGCATCCATATGAAAAAAATTATTTTAACA	*Nde*I
NonOmpAF	CCCATATGAAAGCTACTAAACTGGTACTGGGCAACCCGTATGTTGGCTTTGAAATGGG	*Nde*I
NonOmpAR	CGGAATTCGCTCCCGGAATGCCGTTGTCCGGACGAGTGCC	*Eco*RI

### In silico analysis

To predict the presentation of the signal peptides, we have tested the sequences using two well-known neural network-based programs, including, SignalP (Nielsen et al. 1997; Petersen et al. 2011; http://www.cbs.dtu.dk/services/SignalP) and LipoP (Juncker et al. 2003; http://www.cbs.dtu.dk/services/LipoP/). The first 70 amino acids of N-terminal of each protein were examined in both of the signal peptide predictions. SecretomeP was used for Prediction of non-classical protein secretion (http://www.cbs.dtu.dk/services/SecretomeP). TopPred program a topology prediction of membrane proteins (Claros and von Heijne 1994) was used for hydrophobicity analysis of the examined Lpp-OmpA-PE and Non-OmpA-PE amino acid sequences. Structure of Non-OmpA-PE was predicted by TMRPres2D. MEMSAT3 and MEMSAT-SVM was used for membrane helix prediction (Nugent and Jones 2009; Nugent et al. 2011; http://bioinf.cs.ucl.ac.uk/psipred).

### Plasmid construction

**Fig. 1:**
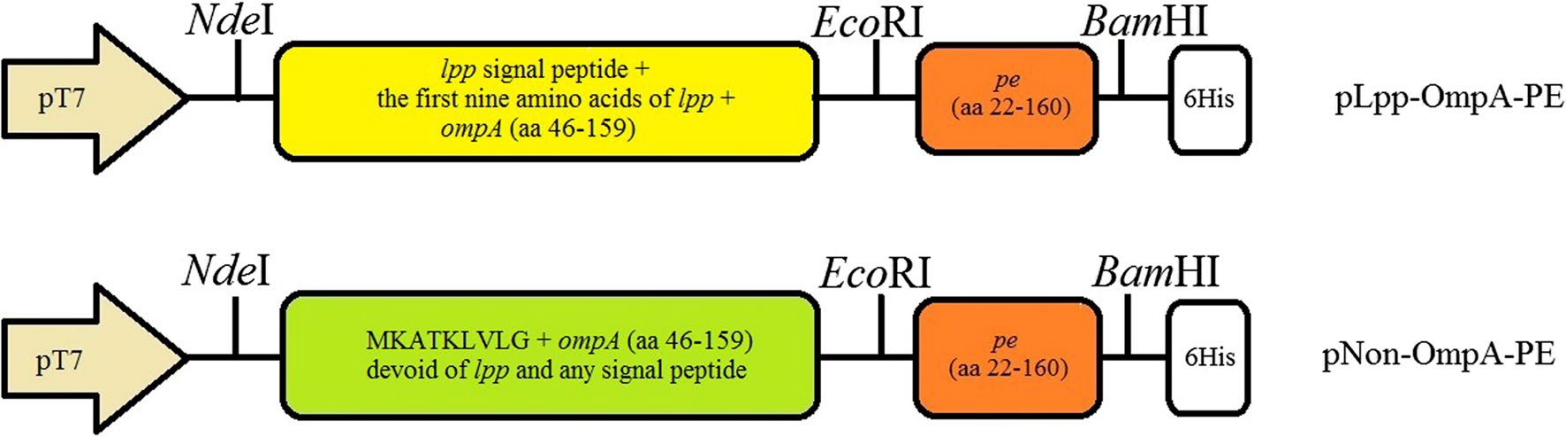
Gene maps of recombinant plasmids harboring truncated *non-ompA-pe* and *lpp-ompA-pe* fusions. Plasmid pET26 was used as parent vector for constructing these fusions

The *lpp-ompA* fusion gene was digested from plasmid pGEM-JR with *Nde*I and *Hin*dIII and then ligated into similarly digested pET-26a to generate pLpp-OmpA. The *pe* gene was PCR amplified from *Haemophilus influenzae* genome using primers PENdeI and PEHindIII, while the restriction enzyme sites *Nde*I and *Hin*dIII were introduced into the forward and reverse primer sequences, respectively. The PCR fragment was subjected to *Nde*I and *Hin*dIII digestion and sub cloned into the corresponding sites of the pET-21b to generate p21PE. The *pe* gene encoding amino acids 22–160 (excluding the signal peptide) was amplified using PCR with primers NcoIpE22 and PEHindIII and then digested by *Nco*I and *Hin*dIII. The insert was ligated into pET-26b that had been digested with similarly enzymes to produce pPE22-160. To create pLpp-OmpA-PE, the *pe* (aa 22-160) was PCR amplified from *Haemophilus influenzae* genome using primers EcoRIpE22 and PEHindIII and digested with *Eco*RI and *Hin*dIII, then ligated into similarly digested pLpp-OmpA. To construct a non-classical plasmid for the expression of the Non-OmpA intracellularly, the *ompA* gene without signal peptide was PCR amplified from *E. coli* genome using primers NonOmpAF and NonOmpAR. The PCR product was digested with *Nde*I and *Eco*RI and then ligated into similarly digested pET-26bto generate pNon-OmpA. The *pe* gene already was PCR amplified and digested with *Eco*RI and *Hin*dIII ligated into similarly digested pNon-OmpAto produce pNon-OmpA-PE. The recombinant plasmids were sequenced to confirm the correct sequence of the inserted genes. Transformation of the plasmid into *E. coli* Dh5a was carried out using the CaCl_2_-mediated transformation (Sambrook and Russell 2001). Figure 1 shows pLpp-OmpA-PE and pNon-OmpA-PE schematically.

### Cell fractionation

Recombinant cells were fractionated according to the method of Sun et al. (Elluri et al. 2014). The cultures were further grown for 18 h after induction and were harvested and centrifuged at 12,000×*g* for 5 min and resuspended in 100 ml of SDS-PAGE sample buffer for the total cell lysates fractions. Osmotic shock was utilized for separation of periplasmic proteins (Libby et al. 1987). Cells harboring recombinant plasmids were harvested and washed in a 20 mM Tris–HCl buffer (pH 8.0) and resuspended in 15 µl of ice cold TES buffer (Tris–HCl 0.2 M, EDTA 0.5 M, Sucrose 0.5 mM) pH 8.0, shaking energetically every two minutes for 20 min. A 22.5 µl of ice cold double-distilled water was added, and the incubation was continued on ice for 30 min. The cells were centrifuged at 16,000×*g* for 20 min. The supernatant was filtered through a 0.22-µm syringe filter and used as the periplasmic fraction for further protein analysis. Then, the cell pellets were resuspended in 10 mM Tris–HCl buffer (pH 8.0), incubated for 1 h on ice and disrupted by sonication (Prasadarao et al. 1996). The lysates were centrifuged at 5000×*g* for 10 min at 4 °C to remove any intact cells after that used a 0.22-µm syringe filter for filtration of supernatant and pelleted by centrifugation at 10,000×*g* for 1 h at 4 °C. The supernatant was saved as the cytoplasmic fraction. For further outer-membrane fractionation, the pellet (total membrane fraction) was resuspended sterilized distilled water. To isolate the inner and outer membranes, N-lauryl sarcosyl was added to the pellet (final concentration of 2%) and was incubated at room temperature and then centrifuged at 15,000×*g* for 30 min. The supernatant was utilized as the inner membrane fraction after dialysis, and it was concentrated by precipitation and the insoluble pellets were resuspended in 20 ml of the 2% SDS buffer as outer membrane protein, and analyzed by using 15% SDS–polyacrylamide gel electrophoresis (Laemmli 1970).

### Western blotting

The presence of Lpp-OmpA-PE and Non-OmpA-PE proteins in the subcellular fractions were confirmed by Western blot using anti-His antibody. Whole-Cell lysates and the soluble fraction and outer membrane fraction were analyzed by primary rabbit anti-6His antibody (Biolegend) at a final 1:1000 dilution in phosphate-buffered saline (PBS) buffer and secondary goat anti-rabbit horseradish peroxidase (Biolegend), at a final 1:2000 dilution in PBS buffer (Sambrook and Russell 2001).

### Immunofluorescence microscopy

Immunofluorescence microscopy was used to investigate the presentation of recombinant proteins on surface display. A 250 µl of recombinant cells harboring Lpp-OmpA-PE and Non-OmpA-PE were harvested and centrifuged at 3500×*g* for 4 min and washed three times with PBS (pH 7.4) supplemented with 3% bovine serum albumin. In the next step, the cells were incubated with the rabbit anti-6His antibody diluted 1:1000 in PBS solution for 1 h at 4 °C. After washing five times with PBS, the cell-antibody complex was incubated 1.5 h at 4 °C with a goat anti-rabbit IgG conjugated with FITC (Sigma, USA) at a dilution of 1:500. For microscopic observation, cells were washed five times with PBS solution to remove unbound anti-6His-FITC antibody, then mounted on microscopic slides and was observed by fluorescence microscopy.

## Results

### Surface display system, protein expression design and construction

Lpp-OmpA was used for developing the novel systems to display heterologous proteins on the bacterial surface. OmpA (aa 46–159) was used to create a novel construction and was compared with Lapp-OmpA and were joined to protein E from *Hemophilus influenzae*. They were analyzed of the secretion capability and transition systems by SignalP and LipoP and SecretomeP software. The bacterial localization was assessed by PSORTb v3.0.2 and the secondary structure and transmembrane helixes were predicted by PRED-TMBB and PSIPRED servers, respectively. Genscrips have done the analysis of negative cis elements and sequences repetition (Zhang et al. 2013). Analysis of amino acid sequence PE with its signal peptide by the program indicated one negative cis elements at the beginning of PE. The element in signal peptide sequence has an adverse impact on PE expression. Therefore, PE was used to devoid the signal peptide (PE 22–160) and join it to pET-26b with *pelB* signal peptide. The expression of protein E containing pelB signal peptide or native signal peptide comparison revealed that in the case of native signal peptide, there was no expression as predicted by Genscripts.

**Fig. 2:**
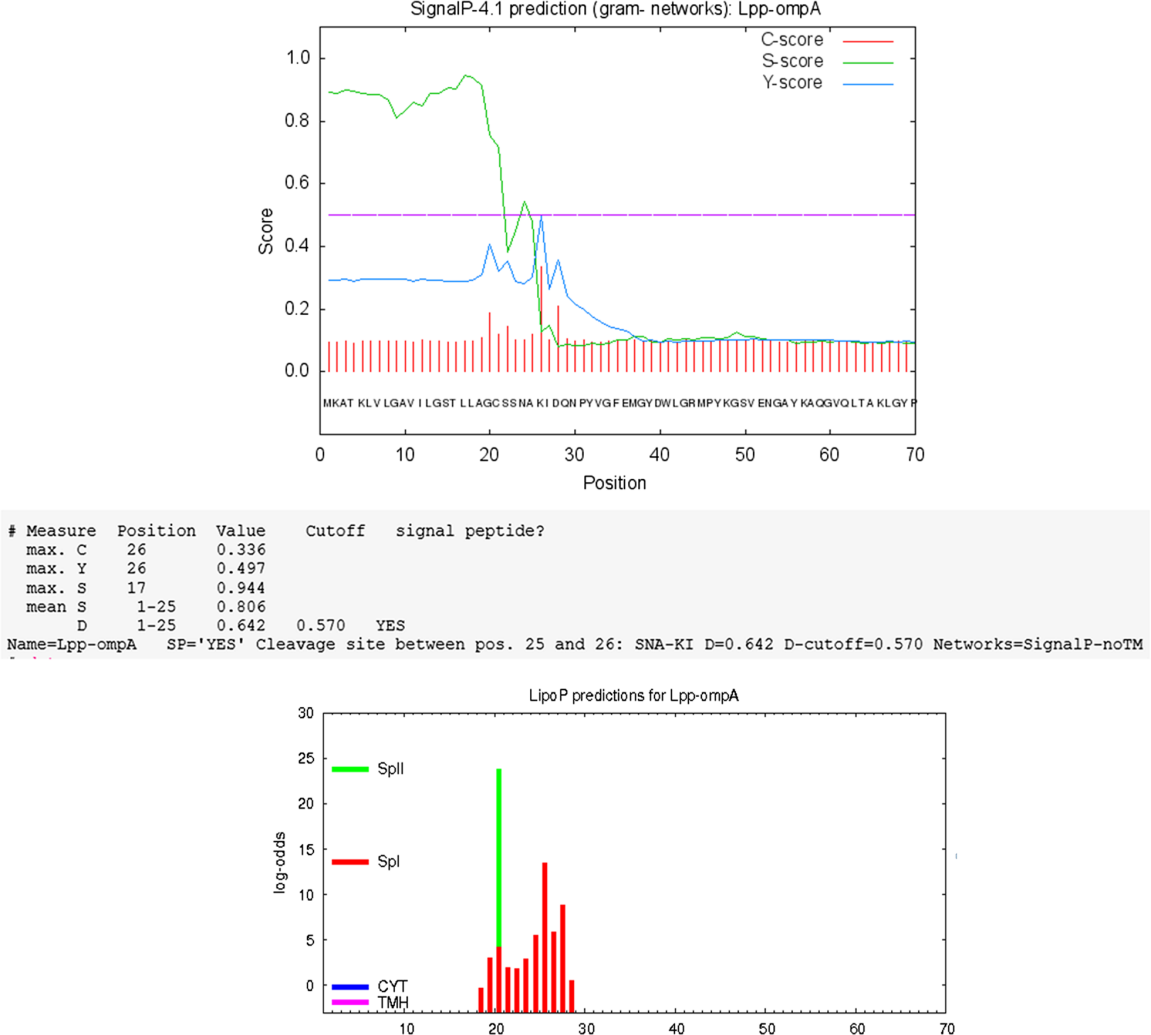
Signal-peptide analyses based on SignalP and LipoP programs performed at http://www.cbs.dtu.dk/services/SignalP/ and http://www.cbs.dtu.dk/services/LipoP/. Lpp-OmpA, the results showed a signal peptide in the Lpp-OmpA-PE

**Fig. 3:**
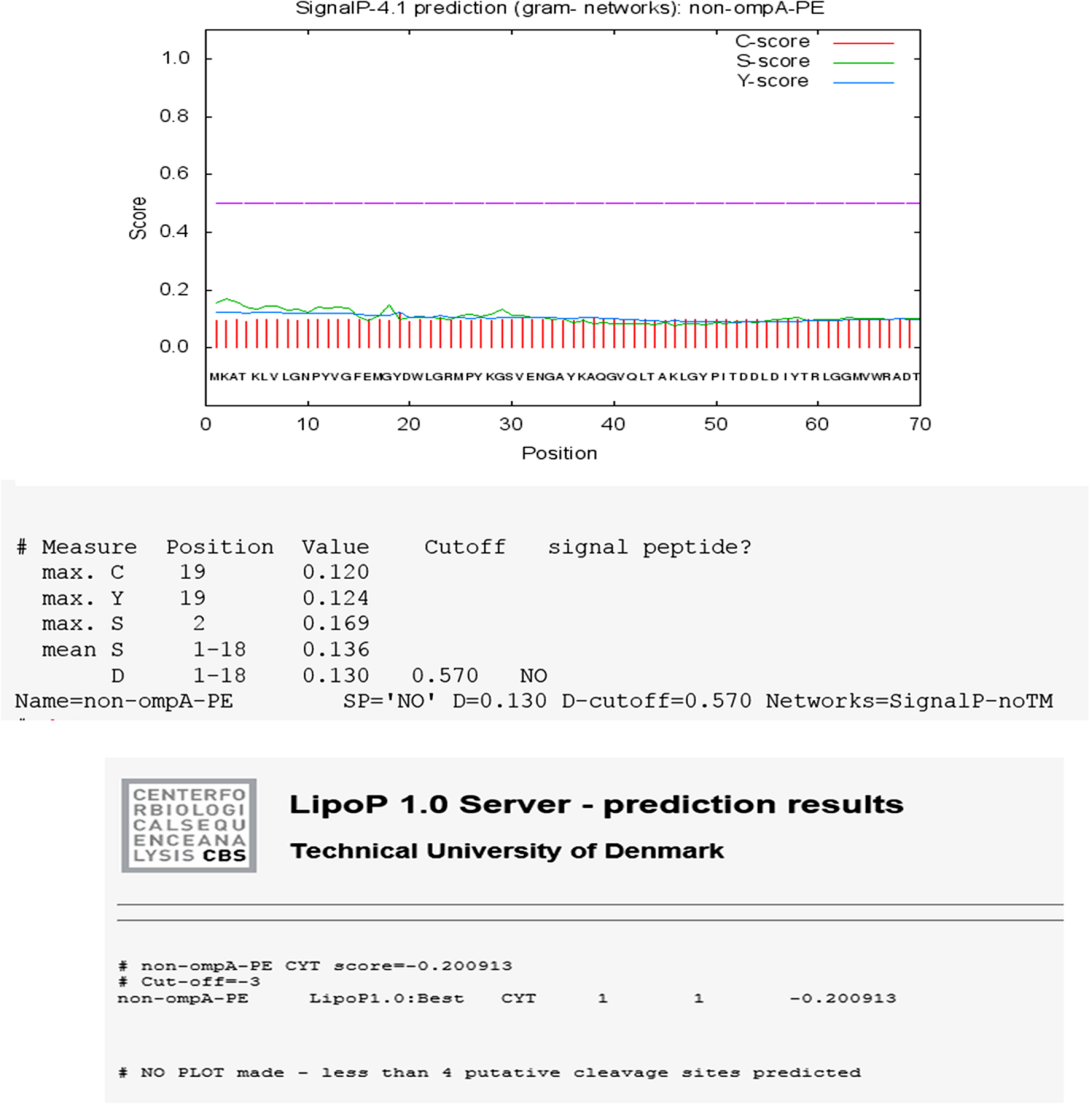
Signal-peptide analyses based on SignalP and LipoP programs performed at http://www.cbs.dtu.dk/services/SignalP/ and http://www.cbs.dtu.dk/services/LipoP/. Non-OmpA, the results showed no signal peptide in the Non-OmpA-PE

**Fig. 4:**
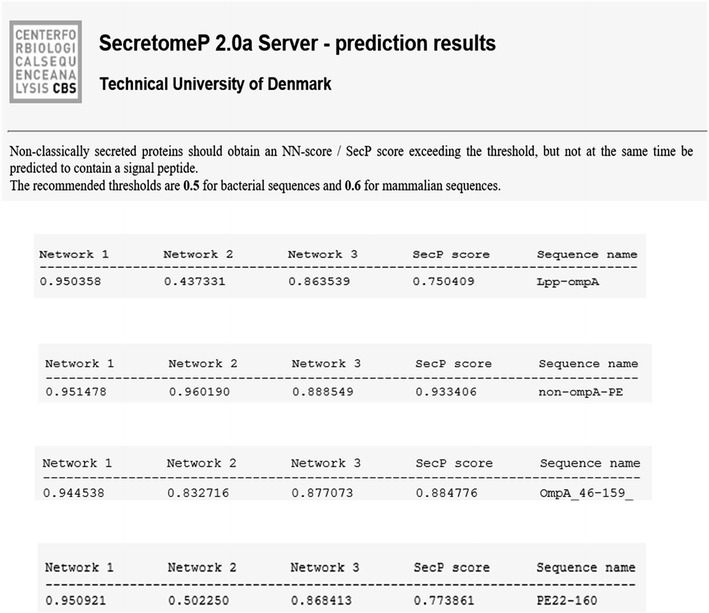
Prediction of non-classical protein secretion by SecretomeP. Amino acid sequences of Lpp-OmpA, Non-OmpA-PE, OmpA (aa 46–159) and PE22-160 were in putted to the SecretomeP was run. The result showed the best score belongs to Non-OmpA-PE

The first step, the recombinant protein sequences Lpp-OmpA, Non-OmpA and PE (22–160) were considered singly by SignalP, LipoP, and SecretomeP. The results indicate capability of them in applying the non-classical pathway for secretion to the surface of *E. coli*. Therefore, the fusions containing Lpp-OmpA-PE and Non-OmpA-PE have been analyzed by SecretomeP. Non-OmpA-PE indicated high validity on the non-classical pathway (Figs. 2, 3, 4). PSORT shown that the fusion protein was located entirely on outer membrane without any signal peptide. The current method leads to the design of Non-OmpA-PE. Also, structure of Non-OmpA-PE in cell membrane was predicted by TMRPres2D.

**Fig. 5:**
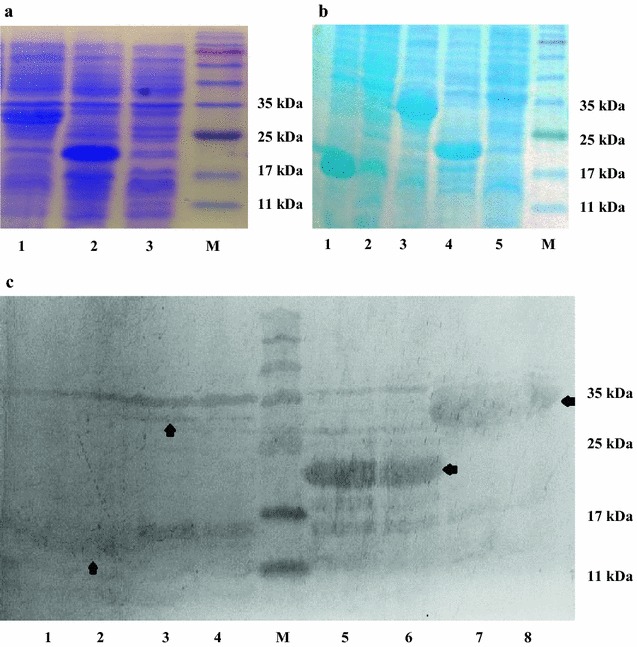
Analysis of the expression of different chimeric proteins. **a** Total cell proteins were analyzed using SDS-PAGE.A: *lanes 1*–*3* represent total cell proteins from *E. coli* BL21 (DE3) harboring pLpp-OmpA-PE, pPE22-160 and negative control, respectively. *Lane M* represents molecular weight markers. **b** The *lane 1* is pNON-OmpA and *lane 2* is pLpp-OmpA. *Lane 3*, *4* and *5* are pNon-OmpA, pPE22-160 and negative control, respectively. The last* lane* is protein marker **c** The presence of chimeric proteins in the total cell proteins from each clone was confirmed by Western blot using anti-His antibody. The bands corresponding to each chimeric proteins were detected in the *lanes 2*–*8*, as Non-OmpA (~16 kDa), Lpp-OmpA-PE (~30 kDa) from clones No. 1 and No. 4, protein E (~23 kDa) from clones No. 3 and No. 7 and Non-OmpA-PE (~30 kDa) from clones No. 3 and No. 5, respectively. *Lane M* represents molecular weight marker and *lane 1* before induction

The construction was developed on merit to keep the original OmpA (aa 46–159) and elimination of Lpp, its signal peptide and addition of nine amino acids to its beginning. Afterward, it joined to PE 22–160 by a linker was located between them. The recombinant plasmids was proved by restriction enzyme digestions, analysis on the agarose gel and DNA sequencing. The expression of the chimeric proteins under the control of the T7 promoter and induction by isopropyl-beta-D-thiogalactopyranoside (IPTG) were confirmed by SDS-PAG and Western blotting with specific anti-6His tag antibody. The expressed recombinant proteins of pLpp-OmpA, pNon-OmpA, pPE, pPE22-160, pLpp-OmpA-PE and pNon-OmpA-PE plasmids were about 16, 23 and 30 kDa, respectively. The results are shown in Fig. 5.

### Cell fractionation and chimeric proteins localization

**Fig. 6:**
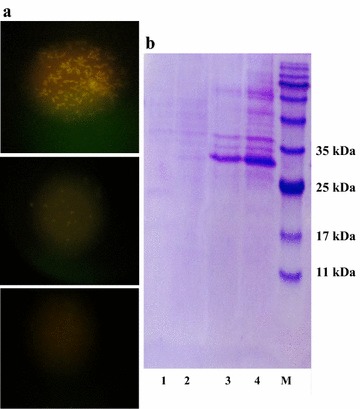
**a** Confirmation of Lpp-OmpA-PE and Non-OmpA—PE fusion proteins displayed on the cell surface. Cells harboring Lpp-OmpA-PE and Non-OmpA–PE were incubated with anti-His-FITC antibody diluted (1:500) for 1 h at room temperature (*top* and *center*) Images show the localization of Non-OmpA-PE and Lpp-OmpA—PE fusion proteins respectively (*down*). No fluorescence signal was detected when the cells were not induced by IPTG. **b** SDS-PAGE analysis of the outer membrane proteins. *Lane 1* is negative control. *Lanes 2*, *3* and *4* represent the outer membrane proteins isolated from *E. coli* BL21 (DE3) harboring pLpp-OmpA-PE, pNon-OmpA-PE, pNon-OmpA-PE (2×), respectively. *Lane M* represents molecular weight marker

To confirm the occurrence of recombinant proteins on the outer membrane, the membrane and soluble fractions were separated and used for SDS-PAGE analysis. The presence of chimeric proteins were illustrated in cytoplasmic and outer membrane fractions. Specific bands corresponding to 30 kDa fusion proteins were successfully detected in the total protein extracts as well as the outer membrane fractions from cells harboring the plasmids pLpp-OmA-PE, and pNon-OmpA-PE. There was no detected signal in non-induced cells (Figs. 5, 6b). The results showed that the cells harboring pLpp-OmA-PE were presented lower quantity than pNon-OmpA-PE.

### Chimers displaying confirmation on the cell surface by immunofluorescence microscopy

The chimeric proteins display on the surface of *E. coli* straightly certified by immunofluorescence staining. An indirect immunofluorescent antibody technique was used to detect surface display on the recombinant host. After fixation, the primary rabbit anti-6His and the secondary goat anti-rabbit FITC antibodies were applied to stain the surface of the *E. coli*. Non-induced *E. coli* cells were employed as a negative control. As mentioned in Fig. 6, *E. coli* recombinant strains express the fusion proteins. The cells harboring Non-OmpA-PE and Lpp-OmpA-PE constructs display Non-OmpA-PE and Lpp-OmpA-PE fusion proteins respectively, while, both the expression and display of fusion protein was augmented in Non-OmpA-PE plasmid harboring cells. Wild-type *E. coli* cells were non-induced by IPTG not showing recombinant proteins on their surface and no fluorescence (Fig. 6a, b). These results demonstrate the successful display of protein E using Non-OmpA as an anchoring motif. In comparison with Non-OmpA, weak fluorescence signals which were observed in *E. coli* cells containing Lpp-OmpA plasmid, suggesting lower surface display efficiency.

## Discussion

The indication of heterologous proteins on the surface of bacteria has been increasingly employed for different uses in microbiology, although many of them are restricted by the translocation potential and the size of chimeric protein or peptides. Lpp-OmpA is a kind of efficient display system that was introduced by Georgiou et al. (1996, 1997). This system consists of a lipoprotein signal peptide and a small part of Lpp that is attached to transmembrane region B3–B7 of OmpA (aa 46–159) and it successfully transfers a variety of proteins onto the bacterial surface. The short Lpp sequence fused to recombinant proteins via fatty acylation exported via the lipoprotein pathway, and inserted into the outer membrane but solely it may not be able to surface-exposed (Francisco et al. 1992; Ghrayeb and Inouye 1984). Structurally, Lpp-OmpA passes five times in outer membrane, and membrane-spanning strands built up-barrel structures that facilitate the translocation of C-terminally attached passenger proteins across the outer membrane (Lower et al. 2005). However, in some research it has been reported that low yield for displaying of some chimerical proteins by this system, which cause of the low translocation efficiency may root in the steric hindrance in the outer membrane (Karami et al. 2014). Lpp-OmpA is an efficient hybrid system that is extremely sensitive to the secondary and tertiary structures of the fusion protein, and it directly influences on its high efficiency (Stathopoulos et al. 1996). Based on the biochemical and biophysical structure of passenger protein, a suitable system must be selected for efficient displaying on the surface.

In bacteria, the classical secretion pathways comprise of Sec, Tat, and lipoprotein signal peptide. The Sec- and Tat-dependent secretion pathways translocate proteins across the inner membrane in Gram-negative bacteria; also extra translocation machinery parts are found in their outer membrane. The N-terminal signal peptide may play a fundamental role in these systems as the label signaling secretion (Dalbey and Kuhn 2012; Mergulhao et al. 2005). Recently, in a system which called non-classical secretion pathway, some bacterial proteins have been found to be secreted without any apparent signal peptide (Muesch et al. 1990; Huang 2012). The non-classical secretion pathway is a new phenomenon that has been identified in cells of many mammals and bacteria, but its functional mechanism has not been so far recognized (Hung et al. 2010; Wang et al. 2013). This pathway causes the transportation of specific proteins outside the cells, particularly in Gram-positive bacteria mainly at stationary phase (Yang et al. 2011). We imagine there is a transmembrane sequence through the membrane in Non-OmpA-PE structure, thus it is entrapped in the bacterial outer membrane instead of secretion in the culture medium. This case is confirmed by the presence of a hydrophobicity charge is visible in some parts of the sequence in hydrophobicity analysis on non-classical sequences by TopPred.

**Fig. 7:**
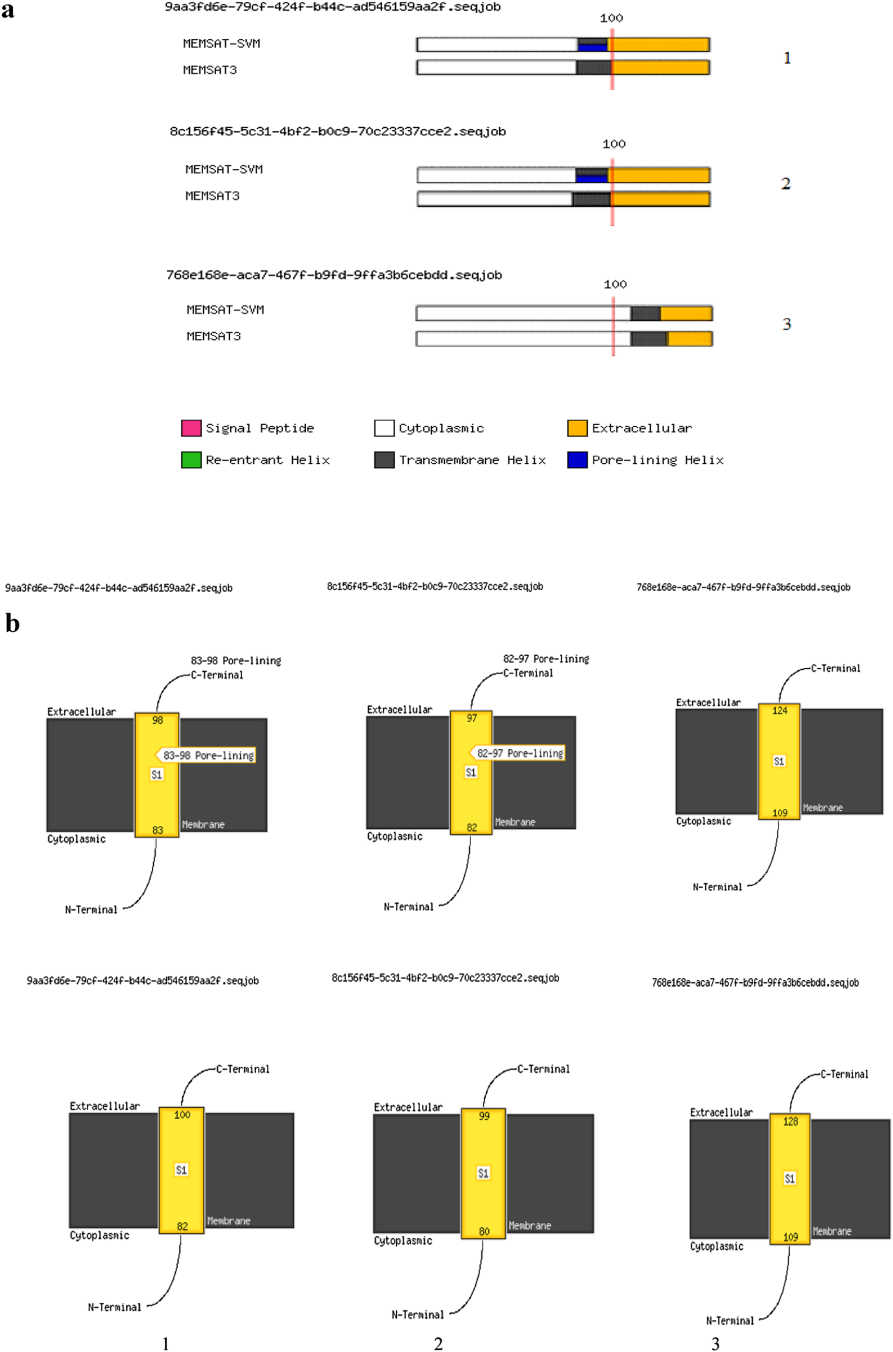
**a** Schematic diagram of the MEMSAT3 and MEMSATSVM predictions for the 150 aa N-terminal domains. **b** The membrane helixes (#83–98), (#82–97) and (#109–124) by MEMSAT-SVM and (#82–100), (#80–99) and (#109–128) by MEMSAT3 are indicated, respectively. **a** MEMSAT-SVM and MEMSAT3 Schematic. *Top* MEMSAT-SVM Prediction and *down* MEMSAT3 Prediction. *1* Non-OmpA-PE and *2* ClyA and *3* Bacillus enolase

Protein E is an outer membrane protein discovered in both encapsulated *H. influenzae* and NTHi. This protein includes a β-sheet formed by six antiparallel β-strands and a long α-helix (Singh et al. 2010, 2013). Also, Protein E is a 16 kDa lipoprotein comprising a signal peptide at the N-terminus, followed by Cys16. Based on the previous studies, protein is transported to the outer membrane accompanied by the addition of lipid chains and removal of the signal peptide by lipoprotein secretion pathway (Tokuda and Matsuyama 2004). The Cys16 residue is thus anticipated to be involved in limitation and functions as an anchor of PE on the outer membrane of bacteria. We proved protein E with its lipoprotein signal peptide has a very low expression probably caused by Cys16 in its signal and helix (aa 1–21), we omitted 22 amino acids at the beginning of protein E and it was substituted with PelB signal peptide. It caused the increase of expression remarkably. Similarly, this effect was verified by the results of analysis with Genscrips (Ronander et al. 2009; Zhang et al. 2013). Protein E is secreted by PelB to periplasmic space, but due to inability to anchor to membrane and display on the surface of *E. coli*. In this study, Lpp-OmpA and protein E were analyzed by SignalP and LipoP and SecretomeP. The predications showed the potential ability of their signal peptide containing sequences despite being odds for non-classical secretion. Then, the signal peptides of Lpp-OmpA (aa 46–159) and protein E from *H. influenzae* were deleted and the potential capacity of each protein for non-classical pathway utilization were analyzed by bioinformatics programs. As the result the capacity did not altered significantly. According to further analysis, fusion of OmpA (aa 46–159) without Lpp and lack any signal peptide with protein E (aa 22–160) from *H. influenzae*, increase the NN sequences core scores of non-classical prediction from 0.43 to 0.96. Based on output of SecretomeP, the secreted proteins of non-classical pathway should acquire an NN-score and SecP score exceeding the threshold about 0.5 for bacterial sequences. But at the same time presentation of a signal peptide shouldn’t be predicted by SignalP. It indicates that the fusion has non-classical secretory potential. Recent studies determined a hydrophobic helix domain (aa 108–126) of enolase N-terminal that might be essential for its secretion, come to the result that the hydrophobic helix domain might be vital for enolase secretion by the non-classical pathway in *E. coli* as in *B. subtilis* (Yang et al. 2014). We obtained similar sequences in the N-terminal of Non-OmpA-PE and also, determined a transmembrane helix by MEMSAT-SVM (aa 83–98) as (aa 83–100) by MEMSAT3 in PSIPRED server. Likewise, their hydrophobicity seemed to be on TopPred, and it indicated a hydrophobic area in that domain (Fig. 7). It offers a pivotal role for hydrophobic regions in N-terminal of studied proteins in secretion by non-classical pathway; however it solely is not adequate. These findings signify that such regions could contain other secretion signals or might indicate a substantial coordination in secretion, but the secretion signals are unknown (Yang et al. 2014).

The results of TMRPres2D software indicate that this structure is properly embedded in the membrane and at the same time similar to case it occurs in *H. influenzae*, PE immunization motifs in region 84-108 are available outside the cellular membrane. In this case, it may show high immunization potential and makes it as an appropriate candidate for the vaccine (Ronander et al. 2009).

A high transcription rate may inhibit the surface display of a recombinant protein as translocation is the restriction step (Rodrigue et al. 1999). The inhibitory effects of overexpression on the translocation pathway have been identified well (Li et al. 2004). In this investigation, high doses of IPTG will induce a high transcription rate; however, the vast quantities of produced protein may not be effectively translocated onto the cell surface. Whereas lipoprotein pathway needs to transport protein after aminoacylation of the N-terminal Cys residue, thus secretion system may create a delay in transportation of recombinant protein onto the outer membrane. Therefore, it is necessary to give enough time to the cell for transport which was done through increasing the induction time and reduction of temperature. Likewise, due to the limitation of transporters in the secretory systems, saturation may negatively affect the transportation of recombinant protein onto the surface of bacteria. The lipoprotein secretion system is responsible for transportation of lipoproteins to the membrane, and transportation should be done to extent without changing membrane structure. For this reason, it is assumed that the cells extremely control transcription and translation of proteins with lipoprotein signals and accordingly, it can explains the reason for under expression in proteins with these signals in some of the previous studies (Karami et al. 2014).

ClyA, a porous protein shows a cytotoxic effect on mammalian cells, transports without any N-terminal signal peptide, at the same time is released from *E. coli* via vesicles that stem from the outer membrane. The ClyA is secreted independently from the five known secretion pathways in Gram-negative bacteria. Thus to achieve the hydrophobic helix, the comparison of Non-OmpA with ClyA in the N-terminal region sequences revealed hydrophobic helix in N-terminal of both structures. It signifies that this domain may be necessary for this pathway (Fig. 7) (Ludwig et al. 2004; Fahie et al. 2013).

These results indicate that the designated structure has the suitable potential for expression of recombinant proteins particularly heterologous membrane proteins onto *E. coli* outer membrane. Also, it may essentially contribute to our perception of quality of function of non-classical secretion pathway.

Briefly, in this survey, Non-OmpA derived from Lpp-OmpA, was employed to display protein E on the cell surface. Non-OmpA was designated for secretion to outer membrane by bioinformatics tools through the non-classical pathway. This research indicates that Non-OmpA is indeed efficient for displaying the recombinant proteins, and it can be assumed as a target in vaccine-related research.

## Additional files

**Additional file 1: Figure S1.** Results of subcellular localization prediction of Non-OmpA and Non-OmpA-PE by PSORTb v.3.0. **Figure S2.** Prediction of presence negative CIS elements in protein E by Genscript. **Figure S3.** Structure of Non-OmpA-PE was predicted by TMRPres2D. **Figure S4.** Comparison of hydrophbicities of the ClyA and Non-OmpA-PE with using membrane proteins prediction program (TopPred) in N-terminal region.

**Additional file 2.** pNon-OmpA-PE. ab1. Sequencing result of pNon-OmpA-PE.

## Abbreviations

PE: protein E
OmpA: outer membrane protein A
Cys: cysteine
NTHI: nontypeable *H. influenzaee*
Lpp-OmpA: lipoprotein outer membrane protein A
INP: ice nucleation protein
Lpp: lipoprotein
IPTG: isopropyl-beta-d-thiogalactopyranoside
FITC: fluorescein isothiocyanate
PBS: phosphate-buffered saline
PCR: polymerase chain reaction
SDS: sodium dodecyl sulfate
Tris: tris(hydroxymethyl)aminomethane
