# Supplementary material for: Designing novel construction for cell surface display of protein E on Escherichia coli using non-classical pathway based on Lpp-OmpA
Source: AMB Express. 2017 Feb 28;7:53. doi: 10.1186/s13568-017-0350-0 (PMC5331024; doi:10.1186/s13568-017-0350-0)
Supplement: Supplementary file 1 — Additional file 1: Figure S1. Results of subcellular localization prediction of Non-OmpA and Non-OmpA-PE by PSORTb v.3.0. Figure S2. Prediction of presence negative CIS elements in protein E by Genscript. Figure S3. Structure of Non-OmpA-PE was predicted by TMRPres2D. Figure S4. Comparison of hydrophbicities of the ClyA and Non-OmpA-PE with using membrane proteins prediction program (TopPred) in N-terminal region. [file 13568_2017_350_MOESM1_ESM.docx]

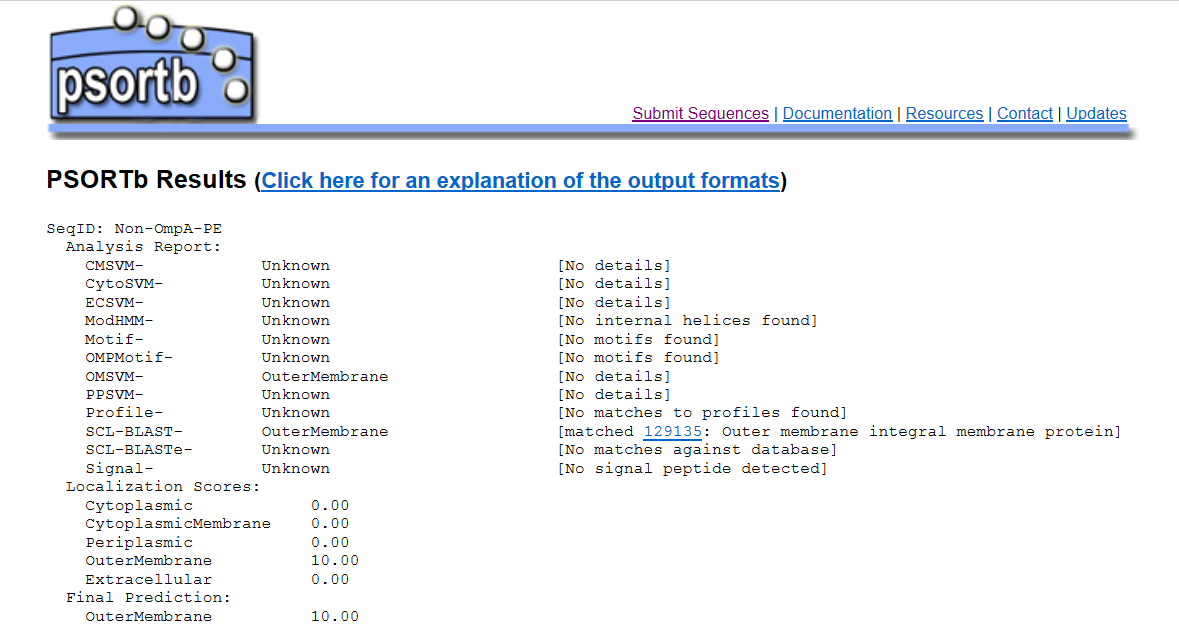

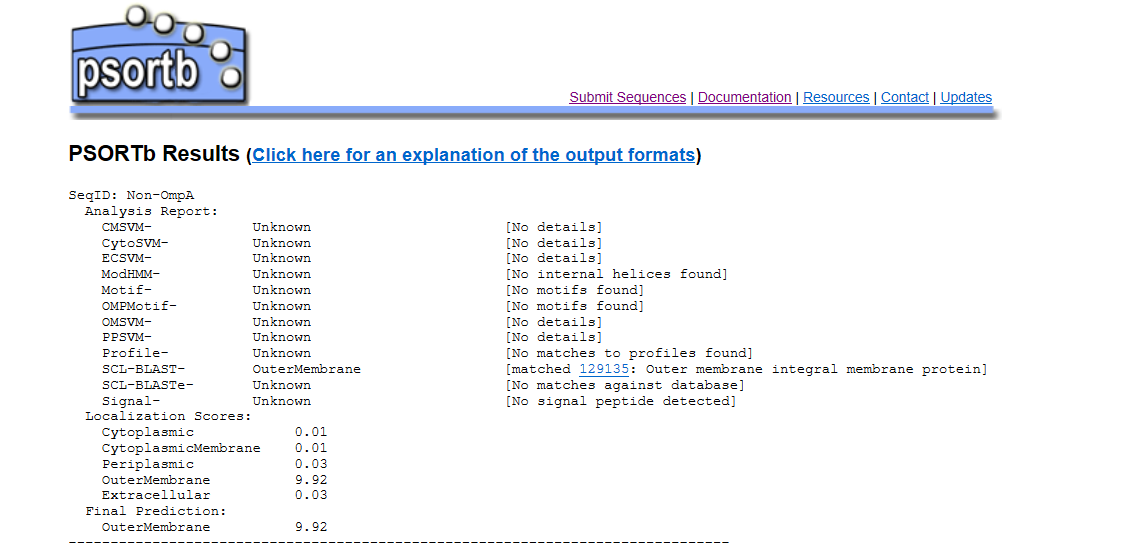


Fig. 1 Results of subcellular localization prediction of Non-OmpA and Non-OmpA-PE by PSORTb v.3.0 <http://www.psort.org/psortb/index.html>


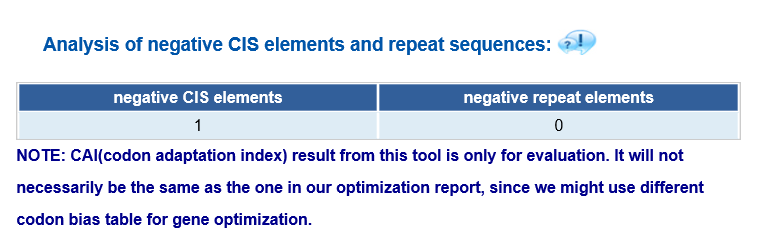


Fig. 2 Prediction of presence negative CIS elements in protein E by Genscript <https://www.genscript.com/>


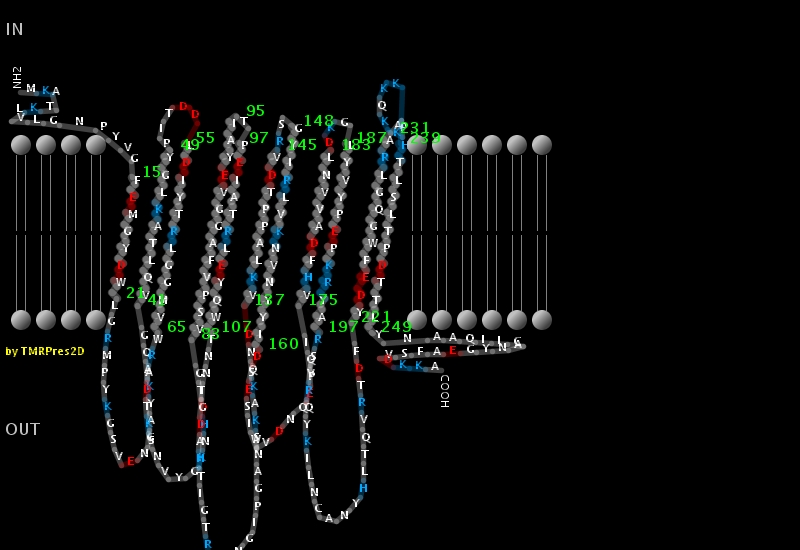


Fig. 3

Structure of Non-OmpA-PE was predicted by TMRPres2D.


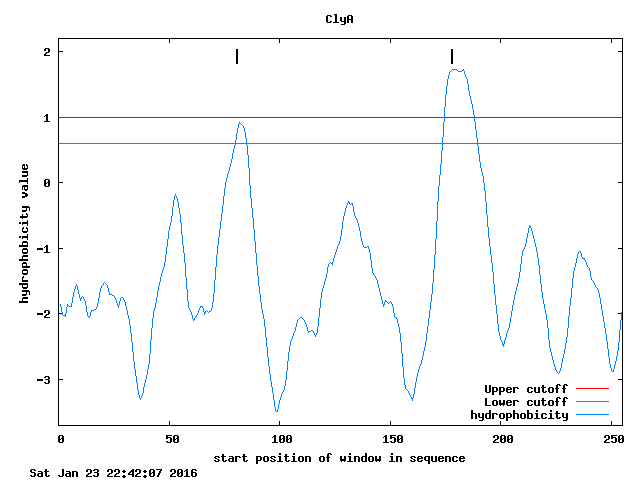

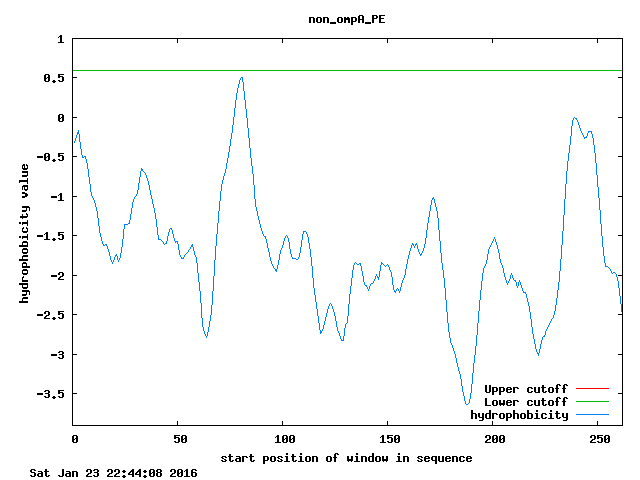


Fig. 4

Comparison of hydrophbicities of the ClyA and Non-OmpA-PE with using membrane proteins prediction program (TopPred) in N-terminal region.
